# Modified Bikini Line Sleeve Gastrectomy (MBLSG): Defining Surgical Landmarks and Clinical Outcomes in a Large-Scale Cohort of 906 Patients

**DOI:** 10.3390/medicina62071326

**Published:** 2026-07-09

**Authors:** Enes Şahin, Mehmet Eşref Ulutaş, Ogün Erşen

**Affiliations:** 1 Department of General Surgery, Faculty of Medicine, Kocaeli University, Izmit 41001, Turkey; dr.enessahin@hotmail.com; 2 Department of General Surgery, Gaziantep Islam Science and Technology University, Gaziantep City Hospital, Şahinbey 27010, Turkey; 3 Department of General Surgery, Bazekol Çiğli Hospital, İzmir 35630, Turkey; ogunersen@hotmail.com

**Keywords:** bikini, bariatric, modified, obesity, sleeve

## Abstract

*Background and Objectives*: Laparoscopic sleeve gastrectomy has emerged as the most widely adopted bariatric procedure in the management of morbid obesity. Despite its efficacy, postoperative scarring at port sites remains a significant aesthetic concern for patients. To mitigate this issue, we developed the Modified Bikini Line Sleeve Gastrectomy technique. This study aims to delineate the surgical steps of the Modified Bikini Line Sleeve Gastrectomy procedure, define the requisite anatomical landmarks, and contribute to the literature by retrospectively evaluating a patient cohort to determine the clinical applicability of this modified approach. *Materials and Methods*: The Modified Bikini Line Sleeve Gastrectomy procedure has been performed at our center since 2020. Stringent patient selection is paramount to the clinical success of this technique. Consequently, specific inclusion criteria necessitate meticulous evaluation during both the preoperative and intraoperative phases. This study delineates the critical aspects of patient selection, provides a comprehensive description of the Modified Bikini Line Sleeve Gastrectomy surgical technique, and analyzes the fundamental intraoperative considerations essential for its successful implementation. *Results*: A total of 906 patients (844 females, 62 males) with a mean Body Mass Index of 40.7 ± 6.6 kg/m^2^ were included. Postoperative surgical site infection occurred in 6.2% of cases, and 0.8% required blood transfusion. At 12 months, the mean total body weight loss and excess weight loss were 35% ± 3% and 86.9% ± 31.2%, respectively. The mean postoperative cosmetic satisfaction score was 4 ± 0.8 (on a 5-point scale). *Conclusions*: These findings constitute the first study in the literature to demonstrate that the Modified Bikini Line Sleeve Gastrectomy technique is safe regarding complication profiles, effective in achieving weight loss goals, and yields high patient-reported cosmetic satisfaction. However, certain clinical presentations may preclude the use of this technique. Specifically, the Modified Bikini Line Sleeve Gastrectomy approach may not be suitable for patients with large hiatal hernias, a history of major upper abdominal surgery, or those with hepatomegaly (left lobe hypertrophy). Furthermore, anatomical constraints such as xiphoid–umbilical, xiphoid–pubic symphysis, and xiphoid–anterior superior iliac spine distances exceeding 25 cm, 36 cm, and 33 cm, respectively along with the presence of a redundant panniculus, represent limiting factors for this modified procedure.

## 1. Introduction

Surgical interventions aimed at the treatment and management of obesity are collectively termed metabolic or bariatric surgery. Currently, bariatric surgery represents one of the most rapidly expanding surgical fields globally. It is estimated that approximately 579,000 procedures were performed in 2014 [1]. Furthermore, in the United States, nearly two million patients underwent bariatric surgery between 1993 and 2016 [2].

Among these modalities, laparoscopic sleeve gastrectomy (LSG) has experienced a surge in popularity, emerging as the most frequently performed bariatric procedure worldwide [3,4]. In the United States alone, LSG was performed in 160,609 patients in 2022, accounting for 57.3% of all bariatric interventions that year. Although initially categorized as a purely restrictive procedure, LSG is now recognized to facilitate weight loss through hormonal modulation specifically by inducing anorexia via the excision of the majority of ghrelin-producing cells located within the gastric fundus [5].

In general, LSG yields excellent weight loss outcomes and facilitates the remission of most obesity-related comorbidities. Compared to other bariatric interventions, such as laparoscopic Roux-en-Y gastric bypass (LRYGB), LSG is associated with lower morbidity rates due to its technical simplicity and preservation of the gastrointestinal anatomy [6]. Consequently, the American Society for Metabolic and Bariatric Surgery (ASMBS) recognizes LSG as the preferred initial approach for high-risk patients [7].

Following massive weight loss, body image dissatisfaction remains a significant psychological challenge for many bariatric patients [8]. Visible abdominal scarring from conventional multi-port laparoscopy can act as a permanent stigma, negatively impacting patient satisfaction, psychosocial well-being, and overall quality of life [9,10]. This is particularly relevant for the predominant demographic seeking these procedures—young female patients—who often place a high priority on postoperative aesthetic outcomes. For this specific population, the profound psychological and cosmetic benefits of concealing surgical incisions within the bikini line robustly justify the adoption of a modified trocar strategy, even though such an approach inherently introduces stricter anatomical selection criteria and specific ergonomic constraints. To mitigate this, various approaches, including reduced-port and single-port techniques, have been developed to improve cosmetic outcomes [11,12]. Among these, the ‘Bikini Line Sleeve Gastrectomy’ (BLSG) was first described by Abdelbaki in 2017 [13], who reported that the technique was safe, feasible, and yielded superior aesthetic results [14]. However, a review of the current literature reveals a paucity of large-scale, multicenter studies validating this technique beyond the original describing institution. Further research is therefore warranted to establish the broader safety profile, reproducibility, and clinical efficacy of this modified approach.

The primary objective of this study is to provide a comprehensive description of the Modified Bikini Line Sleeve Gastrectomy (MBLSG) technique, a refined iteration of the original BLSG approach implemented at our institution. Furthermore, this study aims to establish clinical selection criteria, delineate critical intraoperative landmarks, and report early-term clinical outcomes. To the best of our knowledge, this is the first study to formally describe the MBLSG technique and evaluate its efficacy and safety within a substantial patient cohort.

While inspired by Abdelbaki’s original cosmetic approach, our technique introduces crucial functional modifications. Specifically, the ‘modified’ aspects of our procedure include: (1) an altered diagonal optical axis utilized through a specifically positioned lateral inguinal port, and (2) the complete elimination of a dedicated liver retractor, achieving adequate hepatic clearance solely through synchronized grasper traction vectors. Therefore, this study aims to describe the standardized MBLSG technique in detail and evaluate its safety, feasibility, and technical outcomes in a large-scale, single-center cohort.

## 2. Materials and Methods

### 2.1. Trial Design

This single-center retrospective study analyzed clinical data from patients who underwent the MBLSG procedure between April 2020 and April 2025. A strictly standardized operative protocol was utilized for all patients, with procedures performed by the same experienced surgical team to ensure complete technical uniformity. Before commencing the study, approval was obtained from the Kocaeli University Non-Interventional Clinical Research Ethics Committee (Approval Date: 24 April 2025, Protocol Number: 2025/214). The study was conducted in accordance with ethical rules in accordance with the Declaration of Helsinki. The current study focuses specifically on the surgical technique, anatomical landmarks, and feasibility of the MBLSG modification. It should be noted that the clinical data from this single-center cohort will also serve as the basis for a subsequent, separate comparative analysis (MBLSG vs. Conventional LSG) to evaluate long-term statistical superiority in cosmetic and clinical outcomes.

### 2.2. Surgical Technique

**Figure 1:**
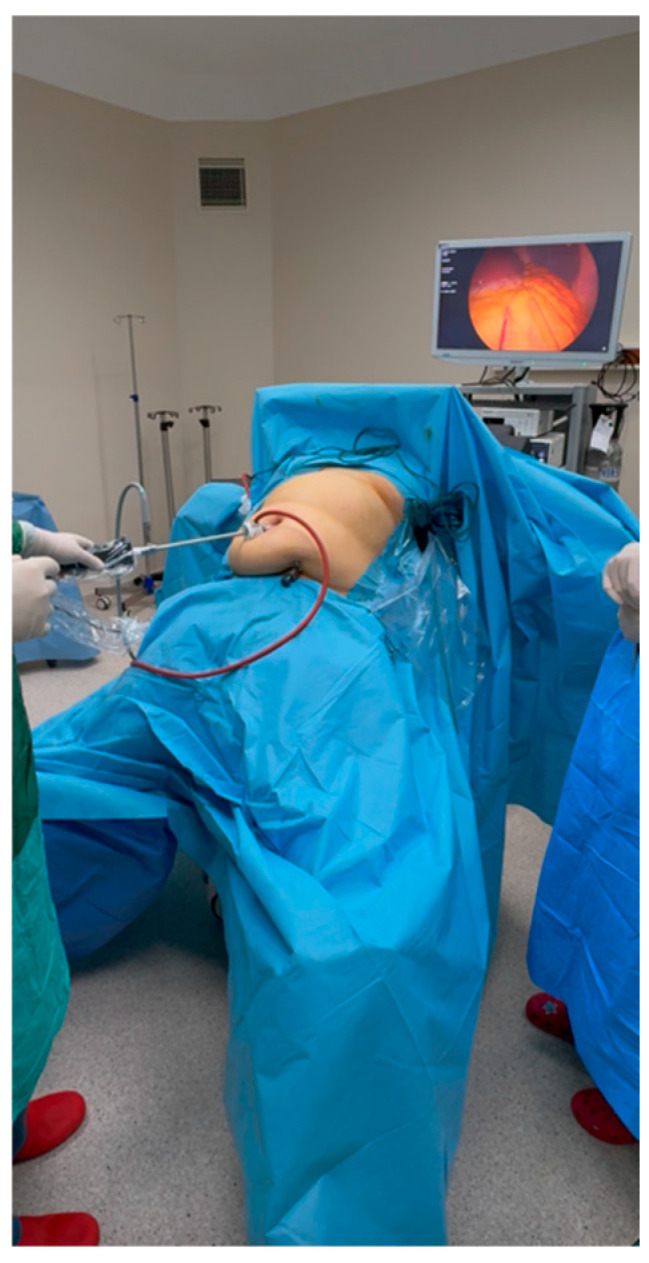
Intraoperative setup and patient positioning in the modified Lloyd-Davies (French) position.

Optimizing patient positioning is the fundamental initial step in any surgical intervention. For the MBLSG procedure, patients are placed in a modified Lloyd-Davies position, characterized by partial knee flexion. The legs are abducted sufficiently to allow the primary surgeon to stand between them (the French position). Both upper extremities are positioned in abduction (Figure 1). In this surgical configuration, the primary surgeon operates from between the patient’s legs, while the camera assistant is positioned to the surgeon’s left, manipulating the laparoscope from an infra-axillary approach. The scrub nurse and the instrument table are situated on the patient’s left side.

The patient must remain in a strictly supine position during the placement of the initial two ports. Maintaining a neutral supine orientation is essential to prevent the displacement of anatomical landmarks. The first step involves the insertion of a 12 mm optical trocar through the umbilicus. Meticulous preoperative disinfection of the umbilical region is mandatory to mitigate the risk of postoperative wound infections; given the recessed nature of this site, inadequate antisepsis may lead to extensive subcutaneous cellulitis.

The presence of a concomitant umbilical hernia may complicate the initial entry. The incision is strategically placed such that one-third is visible externally, while the remaining two-thirds are concealed within the umbilical fold. Due to the inherent density of the umbilical tissue, Veress needle insertion may encounter resistance. Following successful pneumoperitoneum induction via the Veress needle, the 12 mm optical trocar is advanced under direct visualization with concurrent insufflation. It is imperative to identify the precise moment the peritoneum is penetrated, signaled by the visualization of the intra-abdominal cavity. At this juncture, the trocar trajectory must be adjusted from a vertical to a horizontal orientation, directed toward the stomach.

**Figure 2:**
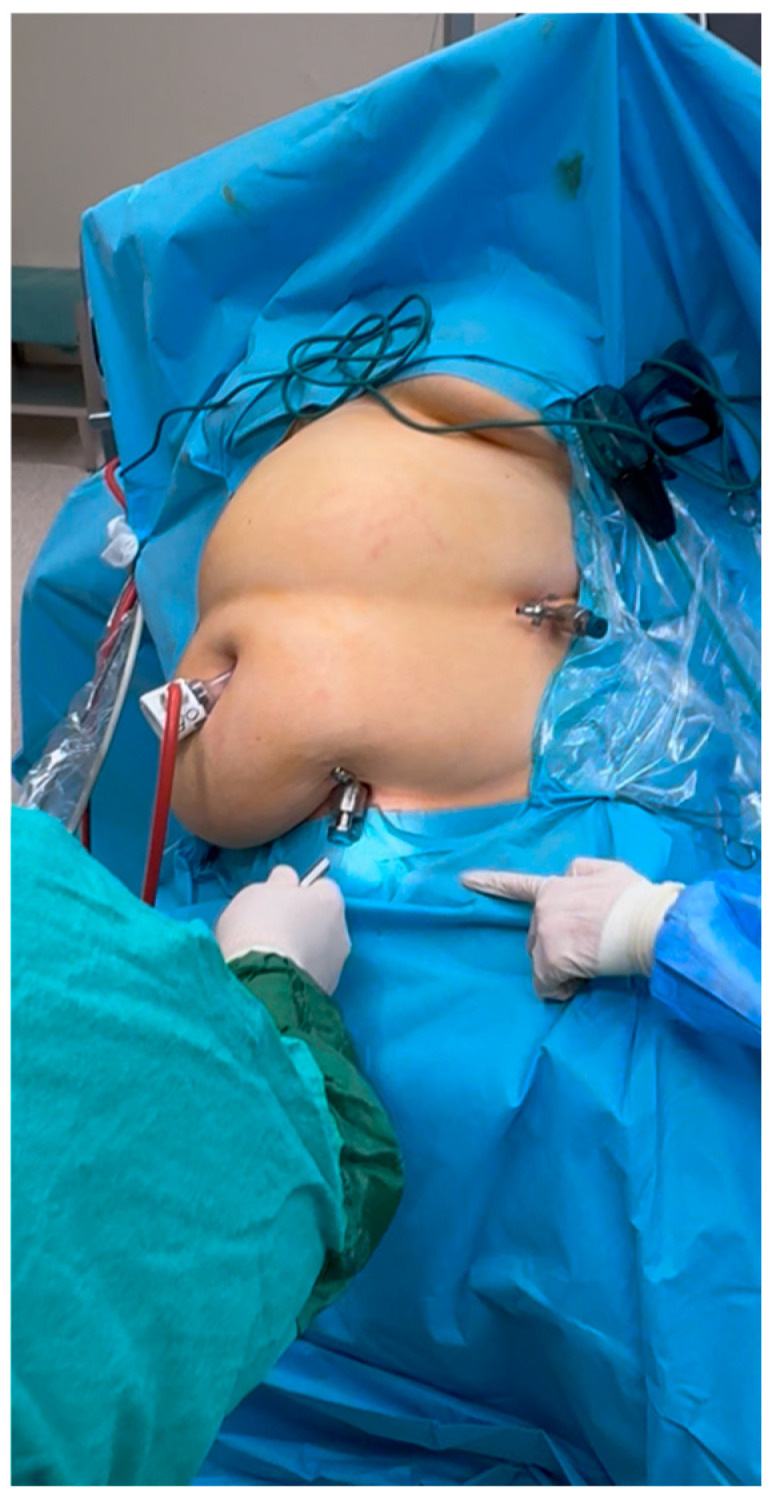
Anatomical landmarks and strategic placement of the three bikini-line trocars.

Following the placement of the umbilical trocar, the second port is established in the left inguinal region using a 10 mm reusable metal trocar. The insertion site is strategically localized at the midpoint of a line connecting the anterior superior iliac spine (ASIS) and the symphysis pubis (Figure 2). For optimal cosmetic outcomes, the incision is placed precisely within the inguinal crease.

This port is introduced via a subcutaneous tunneling technique. The internal peritoneal entry point is situated more cranially than the external skin incision, with a preperitoneal tunneling distance typically ranging from 3 to 5 cm. This oblique trajectory is crucial to mitigate the risk of injury to the inferior epigastric vessels or visceral structures, particularly when instruments are manipulated in the reverse Trendelenburg position. Given that the inguinal crease corresponds to the anatomical projection of the inferior epigastric vessels, direct perpendicular entry poses a significant risk of vascular injury. This risk is minimized by our technique, where the trocar is directed toward the stomach immediately after skin penetration, creating a subcutaneous tunnel.

Although the cutaneous entry point is in the inguinal region, the fascial and peritoneal penetration occurs approximately at the umbilical level. This cephalad displacement is achieved by fully advancing the trocar under direct laparoscopic visualization from the umbilical camera, which also allows for the identification and avoidance of the inferior epigastric vessels. Fully advancing the port ensures that the intra-abdominal portion remains minimal, preventing mechanical interference between umbilical and inguinal instruments. Furthermore, this specific port trajectory ensures that the bowel loops do not obstruct the port during postural changes in the patient.

Following the initial port placements, the patient is transitioned into a steep reverse Trendelenburg position and rotated into a right lateral decubitus orientation to facilitate the insertion of the third trocar. A 5 mm trocar is utilized for this purpose. This postural adjustment is essential to displace intra-abdominal structures, such as the omentum and small bowel loops, thereby providing adequate exposure of the left upper quadrant.

Under direct laparoscopic visualization via the umbilical camera, the third trocar is introduced at the most lateral aspect just below the costal margin. Care must be taken to select an entry point that avoids the retroperitoneum, contingent upon the anatomical positions of the spleen and the descending colon (Figure 2). This port is typically localized inferior to the splenic pole, immediately below the last floating rib. Once the 5 mm trocar is securely positioned, the surgeon can proceed with the subsequent stages of the dissection.

All utilized ports are of standard length; elongated trocars are not required. At this stage, the laparoscope is transitioned to the inguinal port. Given the horizontal trajectory of this trocar, the use of a 30-degree optic is recommended to provide an optimal viewing angle. In our experience, the only minor limitation of this optical orientation is a slight horizontal distortion of the antrum.

The altered trocar configuration in the lower abdomen inherently introduces specific ergonomic challenges, particularly restrictive trocar angulation and potential instrument clashing. As recently highlighted in studies evaluating laparoscopic instrument design and steering capacity, overcoming such distinct geometric limitations demands strict preoperative port-placement planning and advanced surgical dexterity to maintain procedural safety [15].

**Figure 3:**
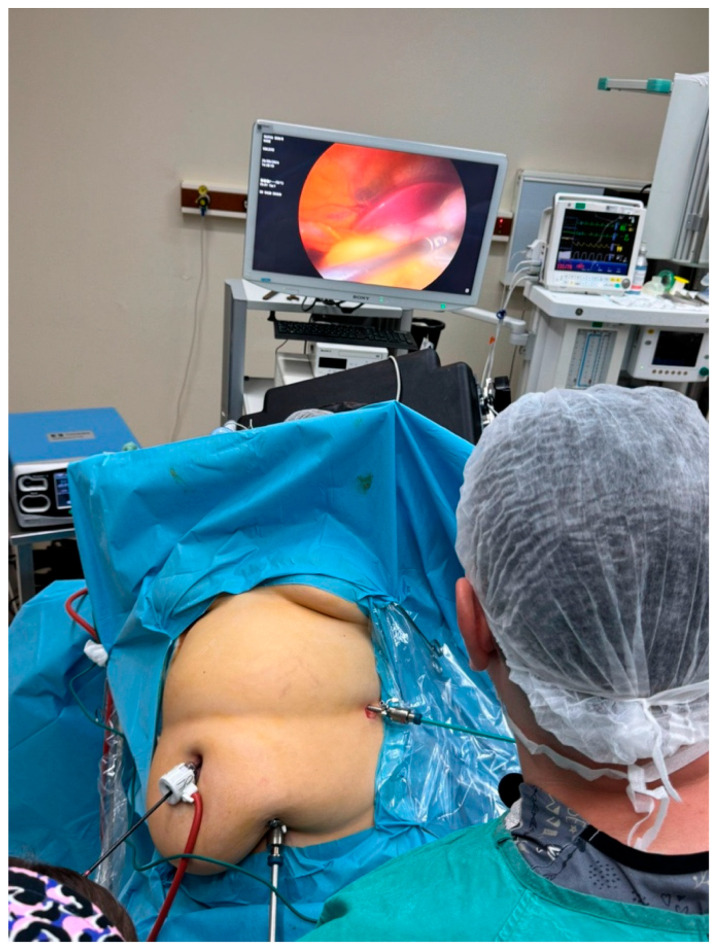
The appearance of surgical instruments and trocars.

Instrument allocation is as follows: a LigaSure™ (Covidien, Mansfield, MA, USA) device is operated via the surgeon’s left hand, while atraumatic graspers are used for traction with the right hand. Elevation and traction are achieved by grasping the greater curvature of the stomach directly, rather than the omentum. Dissection commences toward the spleen to access the posterior gastric space. This initial approach is favored because the umbilical trocar provides an optimal angle for separating the gastrocolic omentum until the fundus is reached. However, as the dissection approaches the fundus, instrument ‘crossing’ may occur, increasing technical difficulty. To mitigate this, instrument positions are swapped: the LigaSure™ is transferred to the right hand to facilitate cardia and fat pad dissection (Figure 3).

**Figure 4:**
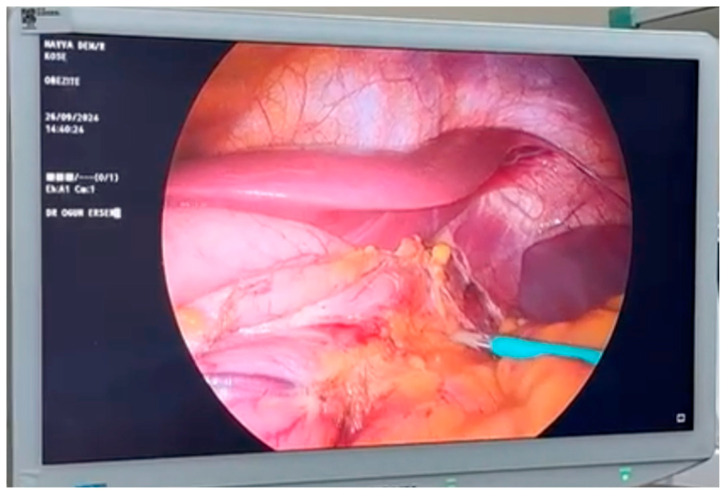
Anatomical view of the operative field after fundus mobilization.

This maneuver enables the left hand to provide traction or elevate the left hepatic lobe as needed. Following the completion of the cardia dissection, the antrum is addressed; maintaining the energy device in the right hand provides a mechanical advantage in this region. The gastrosplenic and gastrocolic omenta are divided using the LigaSure™ device, progressing along the greater curvature in close proximity to the gastric wall. Meticulous exposure of the left crus and the Angle of His is essential. The fat pad at the Angle of His is gently mobilized from its diaphragmatic attachments, ensuring all gastrophrenic connections are severed (Figure 4). Finally, posterior adhesions between the stomach and the pancreas are cleared via sharp and blunt dissection, preparing the stomach for stapling.

**Figure 5:**
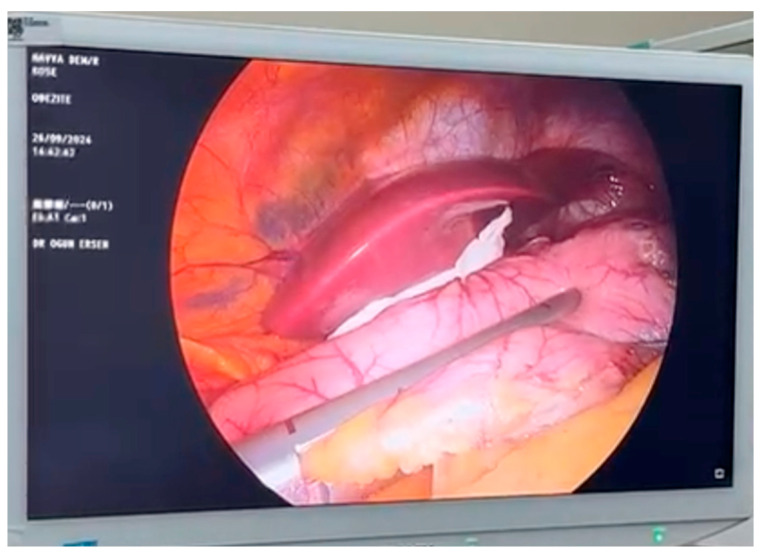
Orientation of the first stapler firing at the antrum, illustrating the angle required from the umbilical port.

At this stage, the gastric sleeve is fashioned over a 36-Fr bougie. The linear stapler is introduced via the surgeon’s left hand (umbilical port), and the gastric transection commences at the antrum, oriented at a sharp angle toward the right. Should the trocar position appear relatively low, cephalad traction is applied to the stomach to optimize the stapler trajectory. The initial firing of the linear stapler occurs 6 cm from the pylorus, utilizing a green or purple cartridge (Figure 5). Gastrectomy is subsequently completed with three to five consecutive firings, depending on the gastric volume.

Following resection, the stapler line is reinforced with sutures. The specimen is then extracted through the umbilical port site. A routine intraoperative leak test using methylene blue is performed in all cases. The umbilical fascial defect is closed with 2-0 absorbable polyfilament sutures utilizing a port-closure needle. To ensure an optimal aesthetic result, the skin is closed with a subcutaneously placed absorbable suture. Drains are not routinely placed in our series.

A structured, step-by-step summary of the operative workflow is provided in Table 1.

To clearly illustrate the technical evolution and specific intraoperative advantages of our approach, we have summarized the key methodological differences between Conventional LSG, the original BLSG, and our proposed MBLSG in Table 2.

### 2.3. Patient Selection

Abdelbaki, who initially described the BLSG technique, identified several contraindications for the procedure, including large hiatal hernias, a history of major upper abdominal surgery, and specific anatomical measurements: xiphoid–umbilical, xiphoid–symphysis pubis, and xiphoid–ASIS distances exceeding 25 cm, 36 cm, and 33 cm, respectively [13]. Drawing upon our extensive clinical experience with the MBLSG modification, we propose additional selection criteria to optimize surgical feasibility.

A primary prerequisite for the MBLSG technique is an appropriately positioned umbilicus. In patients with a low-lying umbilicus, the transition to a reverse Trendelenburg position causes further caudal displacement of the umbilical region, significantly elongating the xiphoid–umbilical distance. While a definitive numerical threshold has yet to be established, our clinical observations suggest that a xiphoid-to-umbilicus distance exceeding one hand-span (approximately 20–24 cm) may compromise the procedure’s technical ease. Notably, performing this measurement in the supine position may yield inaccurate results. Therefore, we recommend that anatomical measurements be conducted preoperatively while the patient is either standing or positioned in a reverse Trendelenburg orientation to reflect the intraoperative reality accurately.

In patients where the xiphoid-to-umbilicus distance exceeds 24 cm, the increased caudal displacement of the umbilical port complicates the initial stapler firing at the antrum due to the excessive working distance. In standard LSG, a right-sided paraumbilical port typically facilitates more direct access to the hiatus through multiple small-angle adjustments. Conversely, in the MBLSG technique, the umbilical trocar is positioned to the left of the pyloric–antrum axis, necessitating a more acute angle for stapler alignment.

This angulation requirement demands intensified traction of the stomach via the third port. However, excessive traction during the initial stapler application carries a risk of gastric torsion (twisting). This occurs because an asymmetrical amount of tissue may be incorporated into the stapler line—specifically, more tissue from the anterior wall compared to the posterior wall. Consequently, in patients with a low-lying umbilicus, this imbalance increases the likelihood of postoperative gastric twisting. Therefore, precise preoperative assessment of the umbilical position is a fundamental prerequisite for the safe and successful implementation of the MBLSG technique.

Another critical consideration is the presence of a redundant or sagging panniculus. In the MBLSG technique, the second trocar is strategically placed in the inguinal region. When the patient is transitioned into the reverse Trendelenburg position, the caudal gravitational pull of the abdominal mass can lead to the frequent dislodgement of this inguinal trocar. Recurrent displacement often results in ‘port laxity,’ causing the trocar to slip out repeatedly and significantly hindering surgical progression.

Because the inguinal port serves as the primary visual access point (camera port) during the mid-stages of the procedure, its frequent dislodgement leads to repeated contamination of the laparoscope upon contact with the visceral structures. Furthermore, due to the subcutaneous tunneling technique used for this port, the fascial entry point is located at a more cephalad level than the cutaneous incision. Consequently, re-establishing the original tunneled path is technically challenging once the port is dislodged. Re-insertion typically necessitates returning the patient to a supine position to realign anatomical planes, which markedly prolongs operative time. Additionally, repetitive trauma from multiple re-insertion attempts can compromise the integrity of the subcutaneous tissue, leading to suboptimal aesthetic outcomes. Based on these observations, we conclude that the MBLSG technique is unsuitable for patients with significant panniculus sagging.

Another pivotal factor influencing patient selection is the anatomical configuration of the left hepatic lobe. This assessment is performed intraoperatively following the insertion of the primary umbilical trocar. Once the patient is positioned in a right lateral decubitus orientation, the feasibility of the MBLSG technique is contingent upon achieving adequate liver retraction. Consequently, an initial laparoscopic exploration is mandatory to evaluate hepatic volume and mobility before proceeding.

In rare instances, patients may present with significant hypertrophy of the left hepatic lobe, which poses a substantial challenge to surgical exposure and may preclude the use of this technique. Conversely, our clinical experience suggests that liver retraction is often more manageable in patients with pronounced hepatic steatosis (fatty liver), as the parenchymal consistency may facilitate displacement. After a comprehensive review of these selection criteria and confirming the patient’s anatomical suitability, the subsequent stages of the MBLSG procedure can be safely executed.

It is important to operationalize how these exclusion criteria were applied. Major clinical contraindications—namely, a redundant sagging panniculus, documented left-lobe hepatomegaly, and the presence of a large hiatal hernia—were applied prospectively from the inception of the study to prioritize patient safety and minimize the risk of intraoperative conversion. Conversely, the strict anthropometric measurements proposed in this study (e.g., specific xiphoid–umbilical, xiphoid–symphysis pubis, and xiphoid–ASIS distances) were derived retrospectively. These precise distances were formalized into an anatomical selection algorithm based on the cumulative ergonomic feedback from the 906 procedures and are proposed herein as standardized guidelines for surgeons adopting the MBLSG technique.

### 2.4. Bailout Rules and Conversion Criteria

To ensure absolute patient safety and minimize learning-curve morbidity, we strictly recommend the following explicit bailout rules for surgeons adopting the MBLSG technique. If any of the following conditions occur, the surgeon must immediately abandon the cosmetic goals and either insert an additional upper abdominal trocar or convert to a conventional multi-port LSG:

Inadequate Liver Retraction: If the left hepatic lobe is excessively hypertrophic or friable, and dynamic retraction using graspers fails to provide clear, stable exposure of the hiatus or the angle of His, a standard epigastric port must be added for a dedicated liver retractor.

Compromised Stapler Angulation: If the altered visual axis or lower abdominal trocar positioning prevents a safe, parallel alignment of the stapler against the calibration tube—particularly risking incisura angularis stenosis or a high-tension staple line—a conventional paraumbilical or subcostal port must be immediately inserted.

Ergonomic Failure due to Panniculus: If a redundant panniculus causes continuous trocar dislodgement, severe instrument clashing, or loss of pneumoperitoneum that disrupts the operative flow, the procedure must be converted to a standard upper-abdominal approach.

Vascular Complications: Any bleeding along the greater curvature or short gastric vessels that cannot be swiftly and confidently controlled via the established lower abdominal ports mandates immediate conversion and the addition of standard working ports.

### 2.5. Data Collection and Outcome Measures

To ensure the reliability of the clinical outcomes, patients who did not attend the 12-month postoperative follow-up visit were excluded from this study. Specifically, 52 patients (5.4% of the initially operated cohort of 958 patients) were lost to follow-up and subsequently excluded. Consequently, the final analyzed cohort of 906 patients represents a 100% completion rate for the 12-month data points. No statistical imputation methods were utilized for missing data, as a complete-case analysis approach was employed.

Demographic and baseline clinical characteristics, including age, gender, height, weight, body mass index (BMI), and American Society of Anesthesiologists (ASA) physical status classification, were retrospectively extracted from institutional records. Postoperative adverse events were systematically reviewed, specifically recording the incidence of surgical site infections, noticeable bruising at the port sites, and any requirement for postoperative blood transfusion. Weight loss efficacy was evaluated at the 12-month postoperative milestone by calculating both the percentage of total body weight loss (%TBWL) and the percentage of excess weight loss (%EWL). Patient-reported cosmetic satisfaction regarding abdominal scarring was evaluated at the 12-month postoperative follow-up visit. This was assessed using a non-validated, institutional 5-point Likert scale ranging from 1 (completely dissatisfied) to 5 (highly satisfied). To minimize investigator bias, the satisfaction scores were collected by outpatient clinical nursing staff independent of the primary surgical team. Due to the single-arm retrospective design of the study, patient blinding was not applicable.

## 3. Results

Since 2020, 906 patients underwent the MBLSG procedure. All 906 procedures were successfully completed using the MBLSG technique; there were no intraoperative conversions to conventional LSG, reflecting the strict adherence to preoperative selection criteria. The study population was predominantly female (n = 844, 93.1%), with a mean age of 34.5 ± 6.8 years. Based on ASA physical status classification, 53.1% of patients were ASA II and 46.9% were ASA III. The mean operative time was 26.9 ± 3.4 min, and the mean length of hospital stay was 2.0 ± 0.8 days.

Regarding postoperative complications, significant bruising at the port sites occurred in 91 patients (10%). The overall surgical site infection (SSI) rate was 6.2% (n = 56). It is crucial to note that all of these were strictly superficial incisional infections (Clavien-Dindo Grade I or II) without any deep fascial dehiscence or intra-abdominal abscess formation. Anatomically, these superficial SSIs were predominantly localized to the paraumbilical port and the suprapubic incisions located within the lower abdominal skin folds. All cases were successfully managed conservatively in the outpatient setting with local wound care and oral antibiotics (empirically or targeted against standard skin flora such as *Staphylococcus* species).

Only 7 patients (0.8%) required postoperative blood transfusion. All of these bleeding events occurred postoperatively and became clinically evident through a significant drop in hemoglobin levels accompanied by tachycardia. Notably, none of the patients required reoperation. All cases were successfully managed conservatively with blood transfusions and close clinical observation. Due to the successful conservative management, the definitive source of bleeding (intraluminal vs. intra-abdominal) was not surgically visualized, though the clinical course suggested self-limiting staple-line bleeding.

The postoperative complication profile was evaluated according to the Clavien-Dindo (CD) classification system. The overall safety profile was highly favorable, where no intraoperative complications occurred. Regarding major bariatric complications, there were no incidences of staple-line leaks (0%), venous thromboembolism (0%), intraoperative conversion to open surgery (0%), or post-sleeve strictures requiring surgical re-intervention (0%). The 30-day readmission rate was 4.5%. According to the CD classification, Grade I complications occurred in 245 patients (27%), which included minor events such as hemorrhage and ecchymosis at the surgical site. Grade II complications were observed in 137 patients (15.1%), encompassing events requiring pharmacological treatment such as surgical site infections or minor transfusions. Notably, regarding more severe complications, no cases were observed for Grades IIIA, IIIB, IVA, IVB, or V, and no mortality was recorded.

Weight loss outcomes at the one-year mark were remarkable, with a mean EWL of 86.9% ± 31.2%. Patient-reported aesthetic satisfaction was high, reflected by a mean cosmetic score of 4 ± 0.8 (Table 3).

## 4. Discussion

In general, LSG is a highly effective and feasible surgical modality for achieving significant weight loss and the subsequent remission of obesity-associated comorbidities. Due to its technical simplicity and the preservation of gastrointestinal anatomy, LSG is associated with lower morbidity rates compared to more complex bariatric procedures, such as laparoscopic Roux-en-Y gastric bypass [6]. Consequently, the ASMBS recognizes LSG as the preferred initial intervention for high-risk patients [7].

The BLSG was introduced by Abdelbaki in 2017 as a specialized approach to minimize postoperative scarring, addressing one of the primary aesthetic drawbacks of conventional port placement [13]. In the preliminary series of 28 selected patients, Abdelbaki demonstrated the safety and efficacy of the technique, reporting favorable cosmetic outcomes. The patient cohort had a mean age of 34.6 ± 3.7 years and a mean preoperative BMI of 42.46 ± 3 kg/m^2^. At the six-month postoperative follow-up, the mean BMI and weight were reported as 28.5 ± 1 kg/m^2^ and 79.8 ± 2 kg, respectively. Notably, the initial study established specific exclusion criteria, deeming patients unsuitable if they presented with large hiatal hernias, a history of major upper abdominal surgery, or anatomical measurements exceeding the following thresholds: xiphoid–umbilical (25 cm), xiphoid–symphysis pubis (36 cm), and xiphoid–ASIS (33 cm) distances [13].

The MBLSG technique described in our study represents a strategic modification of the original procedure introduced by Abdelbaki. While we corroborate the contraindication regarding a xiphoid–umbilical distance exceeding 20–24 cm, our study further delineates critical selection criteria that were not previously addressed in the literature. Specifically, we have identified that a redundant or sagging panniculus serves as a significant limiting factor for this technique. In such cases, the gravitational pull and thickness of the sagging tissue impair the functional stability of the inguinal trocar, leading to recurrent dislodgement and technical compromise. Although an objective, quantitative threshold for panniculus measurement has yet to be established, our extensive clinical experience with a cohort of approximately 1000 patients confirms that this anatomical constraint is paramount for successful implementation.

A critical advantage of the MBLSG technique is achieving adequate hepatic clearance without the need for a dedicated external liver retractor. There is an increasing interest in the surgical community regarding alternative, safe retraction strategies that minimize the number of working ports, as seen in the recent validation of self-retracting intraperitoneal devices [16]. In our cohort, safe liver exposure was successfully managed through optimized patient positioning and selective synchronized grasper traction. However, in order to apply this technique, intraoperative assessment of the left hepatic lobe is paramount. The MBLSG technique is contraindicated in patients whose left hepatic lobe fails to displace laterally when positioned in a right lateral decubitus orientation, or in those presenting with significant left lobe hypertrophy. In such instances, the necessity for a dedicated liver retractor mandates an additional trocar insertion, thereby precluding the application of the modified bikini line approach. These patients should be identified via initial laparoscopic exploration through the umbilical port; if adequate exposure cannot be achieved, the surgeon should transition to conventional LSG or the standard BLSG technique.

To clearly illustrate the technical evolution and specific intraoperative advantages of our approach, we have summarized the key methodological differences between Conventional LSG, the original BLSG, and our proposed MBLSG in Table 1. Notably, the MBLSG not only preserves the superior cosmetic benefits of the original bikini line approach but also optimizes the surgical ergonomic axis from the inguinal port, critically eliminating the need for a dedicated liver retractor.

Following his initial description in 2017, Abdelbaki reported outcomes from a larger cohort of 802 patients in 2023 [14]. The study population had a mean age of 38.9 ± 11 years, a mean weight of 111.2 ± 13 kg, and a mean BMI of 44.1 ± 5.3 kg/m^2^. Complications included postoperative hemorrhage in 11 (1.4%) patients, superficial surgical site infections in 32 (3.9%), and portal vein thrombosis in 2 (0.25%). At the 12-month follow-up, a high patient satisfaction rate of 96.1% was reported [14]. Furthermore, Abdelbaki expanded the clinical utility of this approach in 2024 by reporting its application in gastric bypass surgery [17]. Our results in a series of 906 patients compare favorably with the literature. While Abdelbaki reported a mean weight loss of 79.8 kg at six months, our cohort achieved an impressive 86.9% EWL at 12 months. Furthermore, our surgical site infection rate (6.2%) is slightly higher than the 3.9% reported by Abdelbaki. The observed superficial SSI rate of 6.2% is notably higher than the historical rates reported for conventional upper-abdominal LSG. This higher incidence is anatomically inherent to the bikini line approach in a bariatric demographic. Lower abdominal incisions reside in skin folds that are prone to moisture retention, maceration, and friction, while the umbilicus serves as a known reservoir for bacterial colonization. However, we observed a distinct temporal trend; after introducing a more rigorous preoperative skin preparation protocol—specifically involving meticulous chlorhexidine scrubbing of the umbilicus and strict instructions for postoperative drying of the lower abdominal folds—the incidence of superficial SSIs decreased significantly in the latter half of our cohort.

While not technically identical to the MBLSG approach, Chen et al. in China conducted a comparative analysis between sleeve gastrectomy performed via bikini incisions (n = 72) and the conventional LSG method (n = 90). Their findings indicated no statistically significant differences between the two cohorts regarding operative duration or estimated blood loss. However, the bikini group demonstrated superior scores in overall patient satisfaction concerning scar aesthetics. Furthermore, clinical outcomes—including length of hospital stay, healthcare costs, and postoperative complication rates—were comparable between the groups [18]. Similarly, in our substantial cohort of 1000 patients, no major complications were observed following the MBLSG procedure. There was zero mortality within our series, and therapeutic weight loss targets were successfully attained by all patients.

In the European context, Danys et al. (2021) reported their experience as the first to implement the BLSG technique within the region [19]. They concluded that, when performed with appropriate surgical precision, the technique is safe, effective, and results in nearly scarless and painless postoperative recovery. However, it should be noted that their report was limited to a case presentation, whereas our study provides robust data from a large-scale patient series, further validating the reproducibility and safety of this modified approach.

In conclusion, this study constitutes the first large-scale investigation to formally define the MBLSG technique as a distinct modification of the original BLSG procedure. Beyond providing robust evidence regarding the clinical feasibility and safety of this approach, our findings offer critical insights into the surgical nuances and anatomical constraints essential for successful implementation. By delineating specific patient selection criteria and intraoperative maneuvers, this study serves as a comprehensive guide for surgeons aiming to optimize both functional and aesthetic outcomes in bariatric surgery.

Given the extensive nature of these exclusion criteria—including large hiatal hernias, hepatomegaly, significant panniculus, and strict anatomical measurements—the MBLSG technique is inherently more restrictive than conventional LSG. However, in our routine clinical practice, this modified approach is predominantly requested by female patients seeking to optimize postoperative aesthetic outcomes. Among this specific demographic presenting for primary sleeve gastrectomy, we estimate that approximately 60% to 70% of patients successfully meet these rigorous anatomical prerequisites. Therefore, while these strict criteria limit the universal applicability of the procedure, it remains a highly viable, safe, and cosmetically advantageous option for a substantial majority of the targeted patient population.

Despite the large patient cohort, this study has several limitations. First, its retrospective nature may introduce selection bias. Second, the lack of a direct control group undergoing conventional LSG limits the ability to statistically prove the “superiority” of cosmetic outcomes, although patient satisfaction was high. Third, long-term follow-up beyond one year is required to evaluate the durability of weight loss and potential late complications. Furthermore, the assessment of cosmetic outcomes was based on a simple, non-validated, self-reported 5-point Likert scale rather than a standardized psychometric tool or independent systematic photographic evaluation. Future prospective, comparative studies utilizing validated cosmetic scoring systems are required to substantiate these findings. Finally, since the procedure was performed by high-volume bariatric surgeons, the results may not be immediately generalizable to lower-volume centers without a dedicated learning curve analysis.

While our retrospective data demonstrates promising feasibility, we fully acknowledge that introducing modifications in minimally invasive surgery requires rigorous, stepwise evaluation. As emphasized in recent literature regarding surgical innovation, emerging techniques like the MBLSG should ideally be evaluated through a structured methodology, such as the IDEAL framework, to ensure reproducible long-term outcomes and safety prior to broad clinical adoption [20].

## 5. Conclusions

Since its initial description by Abdelbaki in 2017, the BLSG technique has been established in the literature as a safe and feasible bariatric procedure, evidenced by a substantial series of 802 cases reported from a single institution. Our study represents the first large-scale investigation to validate the MBLSG. Our findings support that this modified approach is safe regarding complication profiles, effective in achieving significant weight loss, and provides highly favorable aesthetic outcomes.

From a technical standpoint, the learning curve for the MBLSG procedure is considered manageable for surgeons already proficient in conventional LSG. The primary challenge during the initial adoption phase involves adapting to the altered intra-abdominal visual axis from the inguinal port and mastering the appropriate traction techniques required for the first stapler firing at the antrum.

However, successful implementation is contingent upon rigorous patient selection. Beyond the established contraindications—such as large hiatal hernias, prior upper abdominal surgery, and specific anatomical thresholds (xiphoid–umbilical > 25 cm, xiphoid–symphysis pubis > 36 cm, and xiphoid–ASIS > 33 cm)—we have identified that a redundant or sagging panniculus and hypertrophy of the left hepatic lobe are also critical limiting factors.

In conclusion, the Modified Bikini Line Sleeve Gastrectomy (MBLSG) is a safe and feasible alternative to conventional LSG when performed by experienced bariatric surgical teams in anatomically appropriately selected patients. By utilizing an altered optical axis and specific traction vectors, the technique successfully eliminates the need for a dedicated liver retractor. While this approach is associated with high patient-reported cosmetic satisfaction in a selected cohort, any definitive comparative superiority versus conventional multi-port LSG remains unproven. Future prospective, randomized controlled studies are strictly warranted to validate these findings and establish definitive long-term comparative efficacy.

## Figures and Tables

**Table 1:** Step-by-Step Surgical Workflow of the Modified Bikini Line Sleeve Gastrectomy (MBLSG).

Procedural Step	Key Technical Details in MBLSG
1. Patient Position	Modified lithotomy (French) position with reverse Trendelenburg to facilitate gravity-assisted downward traction of the viscera.
2. Port Placement	All incisions are confined to the lower abdomen (bikini line). Typically includes right inguinal, left inguinal, suprapubic, and lower paraumbilical ports.
3. Camera Transition	The laparoscope is introduced via the lateral inguinal port, establishing a unique diagonal visual axis directed toward the upper abdomen and hiatus.
4. Dissection Sequence	Mobilization of the greater curvature begins at the greater omentum. Crucially, the left hepatic lobe is elevated dynamically using synchronized grasper traction rather than a dedicated static liver retractor.
5. Stapling	Sequential stapler firing proceeds from the antrum toward the Angle of His. The initial stapler trajectory must be carefully aligned from the lower abdominal ports.
6. Specimen Extraction	The resected stomach specimen is extracted through one of the enlarged lower abdominal port sites (e.g., paraumbilical or suprapubic) to maintain cosmetic concealment.
7. Closure	Fascial closure is performed for port sites ≥ 10 mm. Skin incisions are closed with subcuticular sutures to optimize the aesthetic outcome along the bikini line.
8. Bailout Strategy	If adequate liver clearance or safe stapling trajectories cannot be maintained, the immediate bailout strategy is the insertion of a conventional epigastric port or full conversion to standard multi-port LSG.

LSG: Laparoscopic Sleeve Gastrectomy, MBLSG: Bikini Line Sleeve Gastrectomy.

**Table 2:** Technical and methodological comparison between Conventional LSG, Original BLSG, and Modified BLSG (MBLSG).

Feature	Conventional LSG	Original BLSG	Modified BLSG (MBLSG)
Port Placement	Upper abdomen (epigastric, subcostal, paraumbilical)	Lower abdomen/ Suprapubic area	Lower abdomen with specific inguinal port configuration
Visual Axis	Direct upper abdominal optical axis	Lower abdominal optical axis	Altered diagonal visual axis via lateral inguinal port
Liver Retraction	Dedicated liver retractor required (e.g., Nathanson)	Dedicated liver retractor often required	No dedicated liver retractor required
Cosmetic Outcome	Multiple visible abdominal scars	Scars hidden within the bikini line	Scars hidden within the bikini line
Technical Novelty	Standard established baseline	Enhanced cosmesis	Enhanced cosmesis combined with optimized ergonomic traction without dedicated liver retractors

LSG: Laparoscopic Sleeve Gastrectomy, BLSG: Bikini Line Sleeve Gastrectomy, MBLSG: Modified Bikini Line Sleeve Gastrectomy.

**Table 3:** Demographic and clinical findings.

	MBLSG (n = 906)
Age	34.5 (±6.8)
Gender (Male/Female)	62/844
Height (cm)	161.6 (±6.6)
Weight (kg)	112.4 (±16)
BMI	40.7 (±6.6)
ASA Score	
I	0
II	481 (53.1%)
III	425 (46.9%)
Postoperative Blood Transfusion Requirements	7 (0.8%)
Postoperative Surgical Site Infection	56 (6.2%)
Postoperative Bruising at the Surgical Site	91 (10%)
12-month total weight loss (%)	35 (±3)
12-month excess weight loss (%)	86.9 (±31.2)
Postoperative Cosmetic Score	4 (±0.8)

MBLSG: Modified Bikini Line Sleeve Gastrectomy, ASA: American Society of Anaesthesiologists, BMI: Body mass index.

## Data Availability

The data that support the findings of this study are available from the corresponding author upon reasonable request.
